# Nitrate enrichment alters a *Daphnia*–microparasite interaction through multiple pathways

**DOI:** 10.1002/ece3.925

**Published:** 2013-12-28

**Authors:** Tad Dallas, John M Drake

**Affiliations:** Odum School of Ecology, University of GeorgiaAthens, Georgia, 30606 – 4288

**Keywords:** Environmental change, eutrophication, host-pathogen, Metschnikowia

## Abstract

Nutrient pollution has the potential to alter many ecological interactions, including host–parasite relationships. One of the largest sources of nutrient pollution comes from anthropogenic alteration of the nitrogen (N) cycle, specifically the increased rate of nitrate (NO_3_-N) deposition to aquatic environments, potentially altering host–parasite relationships. This study aimed to assess the mechanisms through which nitrate may impact host–pathogen relationships using a fungal pathogen (*Metschnikowia bicuspidata*) parasitic to crustacean zooplankton (*Daphnia dentifera*) as a tractable model system. First, the influence of nitrate on host population dynamics was assessed along a gradient of nitrate concentrations. Nitrate decreased host population size and increased infection prevalence. Second, the influence of nitrate on host reproduction, mortality, and infection intensity was assessed at the individual host level by examining the relationship between pathogen dose and infection prevalence at ambient (0.4 mg NO_3_-N*L^−1^) and intermediate (12 mg NO_3_-N*L^−1^) levels of nitrate. Host fecundity and infection intensity both decreased with increasing pathogen dose, but increased nitrate levels corresponded to greater infection intensities. Nitrate had no effect on host growth rate, suggesting that hosts do not alter feeding behavior in nitrate-treated media compared with ambient conditions. This study suggests that nutrient enrichment may enhance disease through increased transmission and infection intensity, but that high levels of nitrate may result in smaller epidemics through reduced transmission caused by smaller population sizes and increased pathogen mortality.

## Introduction

Parasites are a structuring force to host populations (Anderson and May 1978; Townsend et al. 2009) and communities (Wood et al. 2007), with the potential to influence ecosystem level processes (Hatcher et al. 2012) and ecological interactions (Refardt and Dieter 2012). However, the influence of parasites on hosts is mediated by environmental variation, either directly through changes in host or parasite demography (Johnson et al. 2007; Hall et al. 2012) or indirectly through modulation of host traits linked to disease or competitive interactions (Kline et al. 2006). An understanding of how environmental variation influences disease is a central goal of disease ecology with implications for understanding epidemic dynamics. Given continued anthropogenic modifications to ecosystems, an understanding of how changes in environmental variables influence community persistence and disease dynamics is a problem of increasing importance.

Nutrient pollution is an exemplary anthropogenic modification to an environmental variable that alters ecosystem structure and function (Bruno et al. 2003; Johnson et al. 2010). Nitrate pollution, particularly, is responsible for changes in animal community composition (Smith 2003), plant productivity (Rabalais et al. 2002), and pH of streams and lakes (Fenn et al. 2003; Sutton et al. 2011). While many of these effects are a result of increased phytoplankton growth, nitrate toxicity also directly influences animal populations and communities (Johnson and Carpenter 2008; Johnson et al. 2010). Nitrate pollution is also of human health concern, as increased nitrate levels observed in many agricultural or industrialized areas (National Research Council 2000) could cause methemoglobinemia, a condition affecting the ability of hemoglobin to carry oxygen (Knobeloch et al. 2000; Camargo and Alonso 2006). Lake ecosystems may be particularly vulnerable to the influences of nitrogen pollution because of their typically longer nutrient residence time relative to terrestrial systems and rapid increase in phytoplankton biomass after nitrogen enrichment (Elser et al. 1990).

Our present understanding of the influence of nitrate on disease in aquatic ecosystems remains limited. One major unanswered question concerns the mechanisms behind an apparent increase in parasite abundance in the presence of increased nitrate (Lafferty 1997; Lafferty and Holt 2003). Field studies on alteration of host–parasite interactions by nitrate have identified several pathways, including nitrate-induced increases in host density (Johnson et al. 2007), increased infection intensity (number of parasite cells per infected host; Johnson et al. 2010), and alterations to habitat use of both host and parasite (Johnson et al. 2010). However, few studies have been able to tease apart the potential causal mechanisms underlying the relationship between nitrate pollution and disease as a result of confounding variables in field systems. Specifically, while the influence of other contaminants such as copper (Civitello et al. 2012) and potassium (Civitello et al. 2012) has been examined, the direct impact of nitrate toxicity on the host–parasite relationship remains largely unexplored. Further, while other studies have tested a small subset (1 or 2) mechanistic pathways through which nutrient pollution potentially acts, this study examines nitrate contamination at three hierarchical levels (and a total of 4 potential pathways), all of which are potentially influenced by increased nitrate concentrations.

We used a model system comprised of *Daphnia dentifera* and a virulent yeast pathogen, *Metschnikowia bicuspidata*, to investigate the impact of nitrate on the host–pathogen relationship on three levels. First, we assessed the impact of nitrate at the host population level, by examining host population size and pathogen prevalence in *D. dentifera* microcosms exposed to the fungal pathogen along a spectrum of nitrate concentrations. Second, we assessed the impact of nitrate on pathogen populations by exposing free-living pathogen spores to a gradient of nitrate concentrations and measuring survival. Lastly, we assessed the impact of nitrate on the host–pathogen interaction at the individual host level by examining the influence of pathogen dose and increased nitrate concentration on infection prevalence, intensity, host growth, and fecundity. These experiments address four possible causal pathways by which nitrate could enhance pathogenic infection: (i) the reduction in host population size through increased mortality or decreased fecundity, (ii) an increase in host susceptibility by stressing host populations, (iii) enhanced infection intensity as a result of nitrate promoting pathogen propagation inside of stressed hosts, or (iv) the reduction in free-living pathogen survival, as fungal spores are sensitive to nitrate when grown in culture (Pitt and Miller 1970). The host population level experiment addresses points (i) and (ii), while the pathogen survival experiment addresses hypothesis (iv). Lastly, the individual level dose–response experiment addresses hypothesis (iii) and lends support for a mechanism of hypothesis (i).

## Methods

### Study system

**Figure 1:**
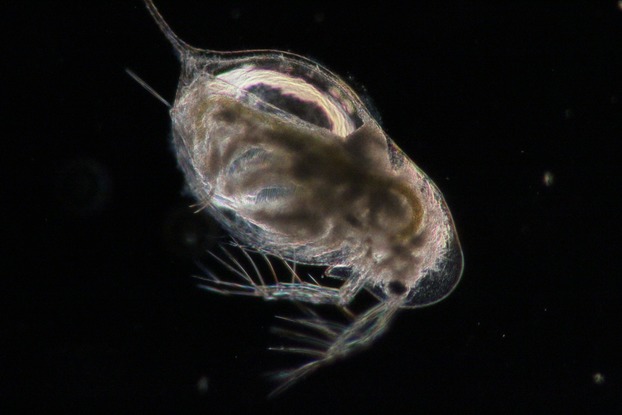
A *Daphnia dentifera* host infected with *Metschnikowia bicuspidata*, a virulent fungal pathogen. The pathogen clogs the gills, heart, and head with needle-shaped spores, making the host appear opaque and white.

Our model system consisted of a single clone of the freshwater cladoceran *D*. *dentifera* reared on a food resource of pulverized blue-green algae *(Spirulina* sp.). *Metschnikowia bicuspidata,* a fungal pathogen used in this study, was cultured in host following the methods of Duffy and Sivars-Becker (2007). The needle-shaped fungus infects hosts after ingestion by piercing the host's gut wall and propagating within hosts, typically leading to host mortality between 10 and 20 days after pathogen exposure (Fig. 1). Nitrate media were produced by dissolving NaNO_3_ in deionized water at concentrations that varied by treatment, autoclaving the solution, and then mixing this sterile solution with filtered pondwater (30-micron filter). Control media were produced by adding sterile deionized water to filtered pondwater.

### The influence of nitrate concentration on host populations

*Daphnia* populations, consisting of 15 isoclonal individuals, were placed in 50 ml of pondwater media supplemented with NaNO_3_ and fed 70 *μ*L of a solution of 0.2 mg *Spirulina* suspended in 100 ml deionized water. A total of 90 populations (for a total of 1350 individuals) were initialized along a gradient of 6 nitrate concentrations (0.4, 2, 4, 8, 16, and 32 mg NO_3_-N*L^−1)^ to investigate the impact of increased nitrate on population and infection dynamics. Populations were inoculated with approximately 10,000 *Metschnikowia* spores (200 spores ml^−1^ of media) at their experimental nitrate levels, allowing nitrate to influence transmission rate. Every other day, half of the experimental media from each population was replaced with fresh media to prevent the buildup of toxic metabolites and reduce the risk of algal contamination. Infection was assessed using a dissecting microscope (20x–50x magnification) 10 days after inoculation. The influence of nitrate on host population size and susceptibility was assessed with Spearman's rank correlations, as a result of the nonlinear response.

### Impact of nitrate on free-living spore survival

The impact of nitrate on free-living pathogen survival was assessed at 7 nitrate concentrations (0.4, 5, 10, 15, 20, 25, and 30 mg NO_3_-N*L^−1)^ using florescent staining to diagnose spore viability at days 0, 1, 20, 40, and 80. Heavily infected individuals were homogenized in sterile pondwater, aliquoted into seven centrifuge tubes, and adjusted to experimental nitrate concentrations. At each observation time, an 18 *μ*L sample was mixed with 2 *μ*L of a 0.125 mg*ml^−1^ propidium iodide solution and allowed to incubate for 15 min at room temperature protected from light. Propidium iodide infiltrates cells with compromised cell walls, which is readily observed using fluorescent microscopy. A total of 20 fields of view at 400x magnification were observed for each sample to quantify the proportion of viable spores at each time step.

The impact of nitrate on free-living spore survival was assessed with a repeated-measures ANOVA, with the proportion of surviving spores as the response variable and nitrate concentration as the treatment factor.

### Impact of nitrate and pathogen dose on infection dynamics and host traits

The influence of nitrate on the relationship between pathogen dose and infection prevalence (i.e., the dose–response relationship) was examined at a single increased nitrate concentration and at an ambient control nitrate concentration. The increased level of nitrate (12 mg NO_3_-N*L^−1^) was chosen to represent a moderately polluted system, reminiscent of some agricultural systems (Camargo and Alonso 2006). A total of 11 pathogen doses were used, starting with 10,240 *Metschnikowia* spores per ml of media as the highest dose and diluting sequentially 10 times by a factor of two in order to obtain the remaining pathogen concentrations (10, 20, 40, 80, 160, 320, 640, 1280, 2560, 5120, and 10240). A total of 462 animals were used to assess the influence of nitrate on the dose–response curve (21 replicates * 2 treatments * 11 dose levels = 462 individuals). To remove maternal effects, we first removed a generation of reproductive females from laboratory stocks. Offspring of this generation were removed and placed into individual tubes and the offspring of this generation were used for experimentation. All hosts were between 24 and 72 h old at the start of the experiment.

*Daphnia* were inoculated together in 200-ml beakers of pondwater media at their respective dose treatments, then each individual was moved to 50-ml test tubes filled with spore-free experimental media the following day to remove any influence of nitrate levels on transmission itself, but allow for nitrate to influence host behavior or immunological responses and/or pathogen fitness. During the inoculation period, half of the normal concentration of food was provided (100 *μ*L *Spirulina* suspension). Infection was assessed using a dissecting microscope (20x–50x magnification) 9 days after inoculation. Intensity was assessed by homogenizing infected *Daphnia* in 200 *μ*L of deionized water and determining spore concentration using a hemocytometer. Offspring were counted and removed from experimental test tubes daily to obtain estimates of fecundity. To determine how nitrate influenced host growth, 40 individuals were placed individually in 50-ml culture tubes of experimental media (20 individuals in 12 mg NO_3_-N*L^−1^ media and 20 individuals in pondwater media containing ambient levels of nitrate). Body length, measured from eyespot to base of tail spine, was assessed for all hosts prior to inoculation and at day 9. The difference between these measurements was used as a measure of growth over the experimental period and was analyzed using a two-sample t-test with a 0.05 significance level.

Two-and three-parameter Weibull and log-logistic models with lower and upper boundaries of 0 and 1 were fit to the dose–response data for nitrate and control media using the *drc* R package (Ritz and Streibig 2005). Two different formulations of the Weibull models were used, referred herein as types “a” and “b”. These formulations differ slightly in their curve fit, as examined by Seber and Wild (1989). These models were competed against one another, with model selection based on negative log-likelihood values. The influence of pathogen dose and nitrate on infection intensity was analyzed with a simple linear regression model. Pathogen dose levels in which only one individual was infected were not considered when fitting the linear model, as these single estimates likely do not represent the true mean infection intensity for that dose treatment.

## Results

### The influence of nitrate concentration on host populations

**Figure 2:**
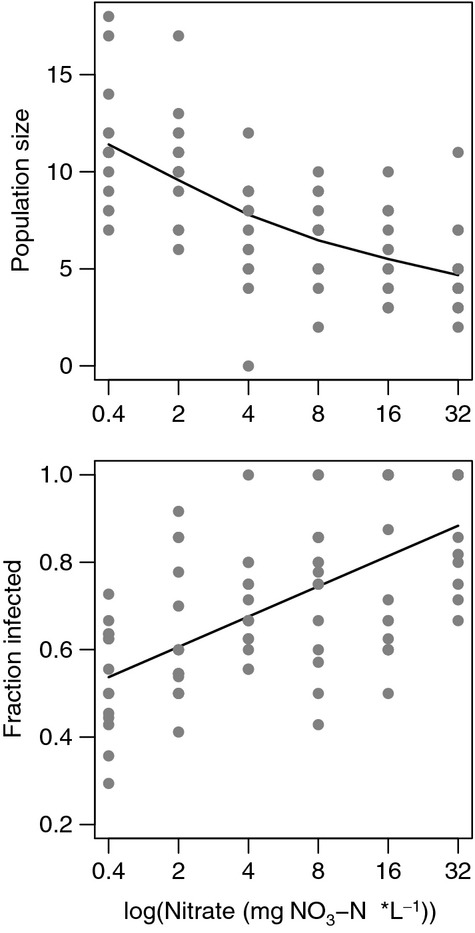
Increased nitrate concentrations resulted in severely reduced host population size (Spearman's rank correlation: *R*_s_ = −0.694, *P *<* *0.0001) and greater infection prevalence (Spearman's rank correlation: *R*_s_ = 0.607, *P *<* *0.0001) after only moderate increases to nitrate concentration. Solid black lines are cubic smoothed splines fit to experimental data (gray points).

Nitrate influenced host populations in two notable ways. First, nitrate caused an increase in infection prevalence, altering prevalence from 53.9% at ambient conditions to 90.7% at the highest nitrate concentration examined. Second, nitrate reduced final host population size (Fig. 2) from an average of 11 individuals at ambient conditions to approximately 5 individuals when exposed to increased nitrate concentrations. Both the effect of nitrate on host population size and the effect of nitrate on infection prevalence were nonlinear. Increased nitrate concentrations were correlated with reduced population sizes (Spearman's rank correlation: *R*_s_ = −0.694, *P *<* *0.0001) and increased infection prevalence (Spearman's rank correlation: *R*_s_ = 0.607, *P *<* *0.0001). Infection prevalence increased to nearly 100% infection prevalence. Meanwhile, population size was reduced drastically in the treatment period between ambient levels of nitrate and 4 mg NO_3_-N*L^−1^, followed by a much slower decrease in host population size (Fig. 2).

### Impact of nitrate on free-living spore survival

**Figure 3:**
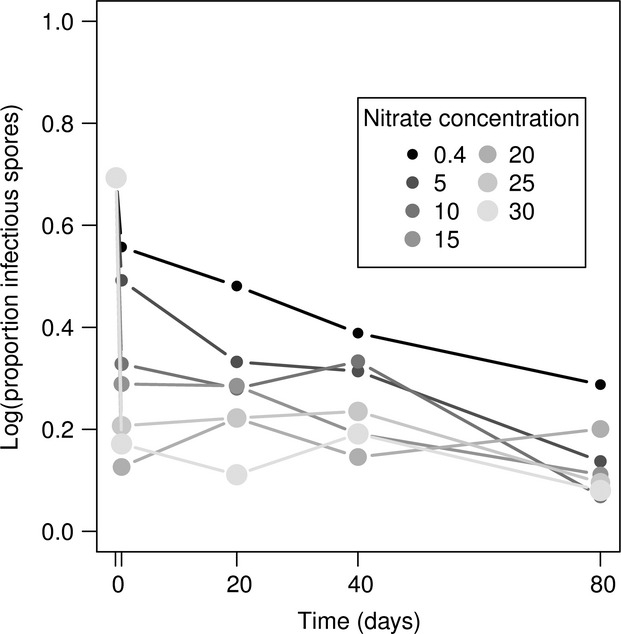
Increased nitrate concentrations greatly reduced free-living pathogen survival after 1 day, suggesting that nitrate could severely reduce the size of the free-living pathogen bank, which is the inoculum source for new infections, and may be responsible for the initiation of seasonal epidemics.

Spore survival was reduced at each time step as a result of increased nitrate concentrations (repeated-measures ANOVA; *F* = 43.22, *P *<* *0.0001). Increased nitrate severely reduced spore survival after 24 h of exposure to nitrate-enriched media, resulting in sharp declines in survival for higher nitrate concentrations followed by asymptotic approach to between 15% and 25% spore survival after 80 days (Fig. 3).

### Impact of nitrate and pathogen dose on infection dynamics and host traits

**Figure 4:**
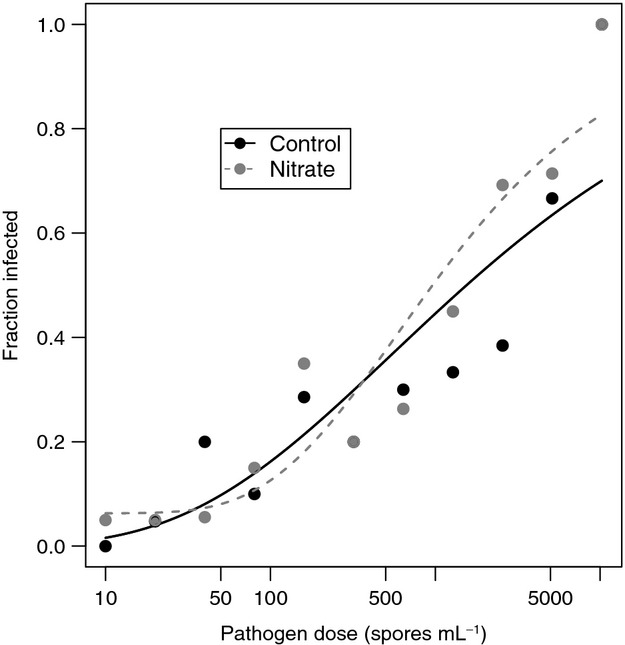
Prevalence increased with increasing pathogen dose in the typical sigmoid fashion, but there was no evidence for a difference in the shape of the dose–response relationship between individuals in control media (solid line; black points) relative to those in media containing increased nitrate levels (dashed line; gray points). Plotted lines are model fits from a three-parameter Weibull dose–response model, with the upper limit fixed at 1, determined to be the best fit model through model selection (Table 1).

**Figure 5:**
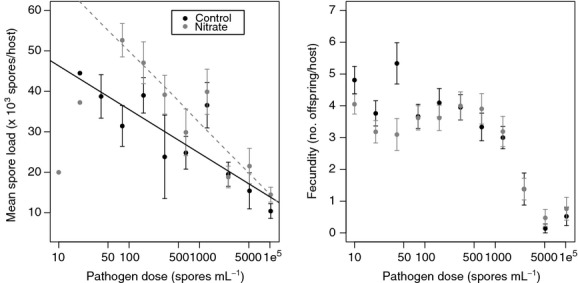
Mean spore load per infected individual declined with increasing pathogen dose (left plot), probably as a result of density-dependent pathogen competition within hosts, while individuals in nitrate media (dashed line) had higher spore loads on average than individuals in the control group (solid line). This relationship is more pronounced at lower (more realistic) pathogen doses. Plotted lines exclude dose treatments with only one infected individual. Pathogen dose decreased host fecundity, even when only considering uninfected hosts. Individuals in both nitrate (gray circles) and control (black circles) treatments did not differ in their fecundity.

Nitrate did not significantly affect the relationship between pathogen prevalence and pathogen dose (Fig. 4). The three-parameter Weibull dose–response model provided the best fits for both nitrate and control dose–response curves based on log-likelihood values (Table 1). Nitrate and pathogen dose both influenced the average spore load of infected individuals (i.e., infection intensity). Infection intensity decreased with increasing pathogen dose, but was enhanced by the presence of increased nitrate levels (Fig. 5). A generalized linear model (Gaussian family, identity link) was used to determine that infection intensity was positively related to nitrate treatment (*t* = 2.39, *P* = 0.032) and negatively related to pathogen dose (*t* = −5.96, *P *<* *0.0001), providing evidence that nitrate causes increased infection intensities consistently across a gradient of pathogen doses. Nitrate treatment and pathogen dose explained 72.3% of the variation in infection intensity. A generalized linear model (Gaussian family, identity link) was used to determine whether host fecundity was altered by increased nitrate or pathogen dose (*R*^2^ = 0.13). Fecundity of uninfected hosts was reduced at higher pathogen doses (*t* = −7.69, *P *<* *0.0001) (Fig. 5), but this relationship was not altered by nitrate (*t* = −1.89, *P* = 0.059). Additionally, host mortality increased as a function of nitrate based on the results of a paired t-test on the number of dead individuals in each pathogen dose class before the experiment was completed (*t* = −3.857, *P* = 0.0039). In the separate group of hosts examined for changes in growth rate, we excluded those individuals that died from analysis and found no evidence that growth rate differed as a function of nitrate treatment (*t* = −0.5618, df = 27, *P* = 0.579).

**Table 1:** Dose-response model fits compared using AIC and log-likelihood (“logLik”). Two variations of Weibull model exist, denoted here as “a” and “b”. Based on log-likelihood values, the three-parameter Weibull model best fit data for both control and nitrate treatments. Bold values indicate the best fit model based on log-likelihood and AIC.

		Control		Nitrate	
Model	Parameters	logLik	AIC	logLik	AIC
Weibull b	3	**11.98**	**−15.96**	**13.12**	−18.23
Weibull b	2	10.27	−14.54	12.79	**−19.58**
Log-logistic	3	10.68	−13.35	12.15	−16.29
Log-logistic	2	8.74	−11.48	11.15	−16.31
Weibull a	3	7.23	−6.45	10.86	−13.72
Weibull a	2	7.20	−8.40	9.20	−12.40

## Discussion

### Major findings

The influence of nitrate pollution was examined with respect to four possible mechanistic pathways discussed in the introduction (hypotheses i–iv). Previous studies have suggested that nutrient pollution has the potential to alter host susceptibility to infection, infection intensity, host population growth, or pathogen survival (specifically in environmentally transmitted pathogens). Through a combination of three experiments performed at three distinct scales (i.e., host population, host individual, and pathogen population), the present study provides evidence that nitrate acts via all four of these pathways, reducing population sizes of susceptible hosts, enhancing both infection prevalence and infection intensity, and reducing free-living spore survival. However, nitrate did not influence host growth or the relationship between pathogen dose and prevalence. Pathogen dose nevertheless decreased host fecundity and infection intensity, which is consistent with previous findings of pathogen-induced life history shifts in hosts (Chadwick and Little 2005; Ohlberger et al. 2011) and decreased infection intensity at higher pathogen doses (Ebert et al. 2000).

### Pathogen dose and infection intensity

The negative relationship between pathogen dose and infection intensity has been observed previously in a *Daphnia*–microparasite system (Ebert et al. 2000). The proposed explanation being that hosts died so quickly at high pathogen doses that the parasites could not propagate within hosts. However, many hosts in our experiment were still alive at the end of the experiment, suggesting that this may not best explain the relationship between pathogen dose and infection intensity. As *Daphnia* respond strongly to cues, it is possible that either hosts recognize the pathogen threat and reduce feeding or the pathogen is so dense that it interferes with the acquisition of algal cells, which would result in reduced feeding (Kirk 1991). Either way, reduced feeding would reduce the number of infectious spores entering the host gut, and this could reduce overall infection intensity.

### Synthesis

At the population level, nitrate reduced host population size and increased infection prevalence. Together, these effects may increase host extinction risk and/or alter host community composition (Fisher et al. 2012). Further, we found that free-living pathogen concentration negatively influenced host fecundity and decreased infection intensity. The decreased infection intensity is not a result of trait-mediated effects, as *Daphnia* were raised and inoculated in ambient pondwater and then placed in experimental media the following day, standardizing the exposure period of host to parasites and removing the potential for nitrate to directly influence transmission rate (as was permitted in the population level experiment). Despite the negative relationship between pathogen dose and infection intensity, increased nitrate resulted in increased infection intensity. Taken together, these results imply that nitrate contamination may result in an increase in environmental pathogen concentration, a corresponding decrease in host fecundity, and a dose-dependent increase in infection intensity. These factors, in turn, contribute more pathogen spores to the environmental reservoir, suggesting that increased nitrate concentrations may result in larger epidemics and reduced host population sizes.

On the other hand, we found a decrease in free-living spore survival with increasing nitrate concentrations. This indicates that large inputs to the free-living pathogen bank as a result of increased nitrate may be masked in natural populations by the influence of nitrate on pathogen survival, as the effect of reduced pathogen survival may counteract the influence of nitrate on spore propagation within hosts. This may become especially important to recurrent epidemics, as epidemics are probably primarily initiated from an environmental reservoir (Duffy et al. 2009). However, the present study examined the influence of nitrate as a stressor, deliberately controlling for the impact of nitrate on other ecosystem properties such as primary productivity. Increases in algal productivity may increase infection prevalence and fecundity (Hall et al. 2007), thereby both increasing epidemic size and reducing the likelihood of host extirpation. While the pathway by which increased nitrate influences pathogen dynamics may be context dependent, it is nevertheless more likely that nitrate will increase epidemic size by causing higher infection intensities and increasing infection prevalence, even if the size of the free-living pathogen bank is reduced, as environmental transmission likely occurs shortly after infected host death.

### Potential implications for epidemics in natural systems

Many environmentally transmitted pathogens cause recurrent epidemics, initiating the epidemic from an environmental pathogen bank. This study suggested that while nitrate may cause higher spore loads in infected hosts, free-living pathogen survival may be reduced at increased nitrate levels. Thus, while nitrate may increase disease severity posttransmission, increased nitrate levels may also reduce the environmental pathogen reservoir, perhaps resulting in a lower likelihood of an epidemic occurring or causing a change in the timing of recurrent epidemics. If pathogen transmission occurs within 24 h of spore liberation from infected hosts, it is likely that nitrate would stimulate epidemics in natural populations. However, if transmission occurs largely through an environmental pathogen source, composed of pathogen spores that have been exposed to increased nitrate for longer than 24 h, nitrate may reduce epidemic size in natural populations. Given the importance of algal resources to host population density and the role of nitrate in promoting primary productivity, it is possible that increased nitrate could result in higher transmission rates through density-dependent transmission. However, it is important to use caution when considering applying these results to natural systems. Our experiments controlled for environmental variability, algal species composition, and other factors that could influence the ecosystem level response to nitrate addition. While our experiments were not intended to mimic natural systems, we do provide an investigation of the mechanisms that underly how nitrate enrichment alters host populations, parasite populations, and host–parasite interactions.

## Conclusions

The present study isolates nitrate as a stressor, providing evidence that nitrate pollution can directly influence host populations and their resident pathogens. Generally, nitrate is likely to influence pathogens in different ways and will likely not always increase disease prevalence or intensity (Johnson et al. 2010). Nitrate deposition in natural settings rarely occurs in isolation of other anthropogenic stressors, making mechanistic field studies difficult. By examining the relationship between an ecosystem level property and infection dynamics in experimental microcosms, we disentangled the mechanisms by which nitrate influences infection at the host population level and with respect to pathogen dynamics. This study represents an important test of the influence of ecosystem level properties on infection dynamics, as laboratory examinations of these interactions are rare (but see Civitello et al. 2012). An useful next step would be to conduct field studies of how environmental factors such as nitrate act both in isolation and in concert with other environmental stressors to influence pathogen dynamics. Together, these will lead to a more complete understanding of how pathogen dynamics are altered in the face of an increasingly variable environment.
